# Gene expression profile of stromal cells from potential metastatic sites in breast cancer patients

**DOI:** 10.1186/1753-6561-7-S2-P54

**Published:** 2013-04-04

**Authors:** Paulo R Del Valle, Maria LH Katayama, Dirce M Carraro, Renato Puga, Eduardo C Lyra, Cintia Milani, Patricia B Rozenchan, Maria M Brentani, Maria AAK Folgueira

**Affiliations:** 1 Department of Radiology and Oncology. Faculdade de Medicina da USP, São Paulo, Brazil; 2 Hospital AC Camargo. Fundação Antônio Prudente, AC Camargo, São Paulo, Brazil; 3 Instituto Brasileiro de Controle do Câncer, IBCC, São Paulo, Brazil

## Background

In breast cancer, there is increasing evidence that stromal cells may influence tumor development in the primary site, as well as in regional and distant metastatic sites. The aim of this study was to compare the expression profile of stromal cells from the primary tumor (PT), lymph nodes metastases (N+) and bone marrow (BM) from breast cancer patients.

## Methods

Fibroblasts primary culture was established from 11 breast cancer patients. Expression analysis was evaluated in PT (n=4), N+ (n=3) and BM (n=4) through a customized cDNA microarray platform (4,800 ORESTES) analyzed by SAM (TMEV; FDR 0%) and functional analysis was performed using DAVID. Technical validation was performed in 6 samples and biological validation was performed in fibroblasts obtained from others 25 patients as evaluated by RT-qPCR.

## Results

We observed 267 differentially expressed genes among fibroblasts obtained from the three different sites, which appropriately clustered them in accordance with their origin. Although differences between PT vs. N+ were represented by 20 genes, differences between PT vs. BM and N+ vs. BM were more significant (235 and 245 differentially expressed genes respectively). DAVID analysis revealed that these cells differed in many functions including those related to development. Among differentially expressed genes were NOTCH2 was confirmed less expressed in PT vs. N+ and USP16 confirmed less expressed in PT vs. BM.

## Conclusion

In breast cancer patients, stromal cells obtained from different origins present a differential gene expression profile.

## Financial Support

FAPESP (process number 2009/10088-7) and CAPES.

